# Is Mate Choice in Humans MHC-Dependent?

**DOI:** 10.1371/journal.pgen.1000184

**Published:** 2008-09-12

**Authors:** Raphaëlle Chaix, Chen Cao, Peter Donnelly

**Affiliations:** 1 Department of Statistics, University of Oxford, Oxford, United Kingdom; 2 Unité d'Eco-Anthropologie, CNRS UMR 5145, Musée de l'Homme, Paris, France; 3 CAS-MPG Partner Institute for Computational Biology, Shanghai, China; 4 The Wellcome Trust Centre for Human Genetics, University of Oxford, Oxford, United Kingdom; University of Chicago, United States of America

## Abstract

In several species, including rodents and fish, it has been shown that the Major Histocompatibility Complex (MHC) influences mating preferences and, in some cases, that this may be mediated by preferences based on body odour. In humans, the picture has been less clear. Several studies have reported a tendency for humans to prefer MHC-dissimilar mates, a sexual selection that would favour the production of MHC-heterozygous offspring, who would be more resistant to pathogens, but these results are unsupported by other studies. Here, we report analyses of genome-wide genotype data (from the HapMap II dataset) and HLA types in African and European American couples to test whether humans tend to choose MHC-dissimilar mates. In order to distinguish MHC-specific effects from genome-wide effects, the pattern of similarity in the MHC region is compared to the pattern in the rest of the genome. African spouses show no significant pattern of similarity/dissimilarity across the MHC region (relatedness coefficient, *R* = 0.015, *p* = 0.23), whereas across the genome, they are more similar than random pairs of individuals (genome-wide *R* = 0.00185, *p*<10^−3^). We discuss several explanations for these observations, including demographic effects. On the other hand, the sampled European American couples are significantly more MHC-dissimilar than random pairs of individuals (*R* = −0.043, *p* = 0.015), and this pattern of dissimilarity is extreme when compared to the rest of the genome, both globally (genome-wide *R* = −0.00016, *p* = 0.739) and when broken into windows having the same length and recombination rate as the MHC (only nine genomic regions exhibit a higher level of genetic dissimilarity between spouses than does the MHC). This study thus supports the hypothesis that the MHC influences mate choice in some human populations.

## Author Summary

There has been a longstanding hypothesis that selection may have led to mating patterns that encourage heterozygosity at Major Histocompatibility Complex (MHC) loci because of improved immune response to pathogens in the offspring of such matings, and, indeed, this has been observed in several model systems. However, in humans, previous studies regarding the role of the MHC in mate choice or preference, both directly in couples and also indirectly in “sweaty T-shirts” experiments, have reported conflicting results. Here, by using genome-wide genotype data and HLA types in African and European American couples, we test whether humans tend to choose MHC-dissimilar mates. This approach allows us to distinguish MHC-specific effects from genome-wide effects. In the African sample, the patterns at MHC loci is confounded by genome-wide effects, possibly resulting from demographic processes relating to the social organization of this population, and no tendency to choose MHC-dissimilar mates is detected. On the other hand, the sampled European Americans appear to have favoured MHC-dissimilar mates, supporting the hypothesis that MHC influences mate choice in some human populations. Thus, this study suggests that, in some cases, humans may rely on biological factors, in addition to social factors, when choosing a mate.

## Introduction

In vertebrates, several studies have revealed that highly polymorphic genes within the Major Histocompatibility Complex (MHC) may have a role in mate choice. In particular, it has been shown that MHC genes influence individual body odor in mice and rats [1]–[7] and that mice prefer MHC-dissimilar mates e.g. [8]–[11], and [12] for a review. Evidence for MHC-disassortative mating was also found in sand lizards [13]. Studies in fish (and in particular Arctic charr) have shown their ability to discriminate the odors of similar and dissimilar MHC siblings [14], and shown that salmon prefer MHC dissimilar mates [15] while female sticklebacks choose a mate in order to complement their own set of MHC genes and to optimize the number of different alleles in their offspring [16]. Complex MHC-based mate choice was also observed in birds [17],[18]. The MHC is the most important part of the genome with respect to immunity [19] and such MHC-based mate choice could increase or optimize the number of non-self antigens that future offspring can recognize and thus increase their resistance to pathogens [12],[20],[21]. It could also have contributed to the extraordinary polymorphism observed at MHC loci [20].

On the other hand, in humans, the role of the MHC in mate choice is very controversial. Ober et al studied classical HLA types for 400 couples from the Hutterite community and found significantly fewer HLA matches between husbands and wives than expected when taking into account the social structure of Hutterites [22]. On the other hand, no evidence of MHC-based mate choice was found in a study of 200 couples from South Amerindian tribes [23]. In a less direct way, other studies have focused on odor preferences: in “sweaty T-shirts experiments”, in which females were asked to smell T-shirts worn by different males, it was shown that females significantly prefer the odor of T-shirts worn by MHC-dissimilar males, although such preference was not found among females taking the contraceptive pill [24],[25]. However, in another sweaty T-shirts experiment, in which males where chosen from a different ethnicity from the females and females were not aware of the nature of the smell (contrary to the two previous studies), females significantly preferred the odor of males having a small number of HLA alleles matching their paternal inherited alleles than the odor of males having fewer matches [26]. Although it has not been established that odor preference is a key factor in mate choice, such studies support the hypothesis that humans are able to discriminate MHC types of potential mates through odor cues and that humans may use such information when choosing a mate. However, the lack of congruence between these studies means that there is still uncertainty as to whether MHC variation influences mate choice in humans, and to what extent. The availability of genetic variation data at genomic scales now allows careful assessment of this question. Crucially, it allows us to distinguish MHC-specific effects from genome-wide effects.

In this study, we tested the existence of MHC-disassortative mating in humans by directly measuring the genetic similarity at the MHC level between spouses. These data were extracted from the HapMap II dataset, which includes 30 European American couples from Utah and 30 African couples from the Yoruba population in Nigeria [27]. Our analyses are based on HLA types and on 9,010 Single Nucleotide Polymorphisms (SNPs) densely distributed across the MHC. Moreover, in order to control for genome-wide effects, we compared the pattern observed in the MHC region to patterns assessed in the rest of the genome, using 3,214,339 SNPs.

## Results

The genetic similarity at a given genetic variant for a given couple *c* was measured using a relatedness coefficient *R*, defined as a ratio of probabilities of identity in state *R* = (*Q_c_*−*Q_m_*)/(1−*Q_m_*), where *Q_c_* is the proportion of identical variants between the two spouses and *Q_m_* is the mean proportion of identical variants in the sample (that is, averaged over all possible pairs of individuals). This coefficient, combined over the genetic variants in a region or across the genome, allows an assessment of whether spouses are more genetically similar or dissimilar than random pairs of individuals. Significance was assessed by permuting individuals between couples. All *p*-values below are two-sided. Positive values of *R* indicate genetic similarity between spouses and negative values indicate genetic dissimilarity between spouses, relative to random mating in the sample.

**Figure 1:**
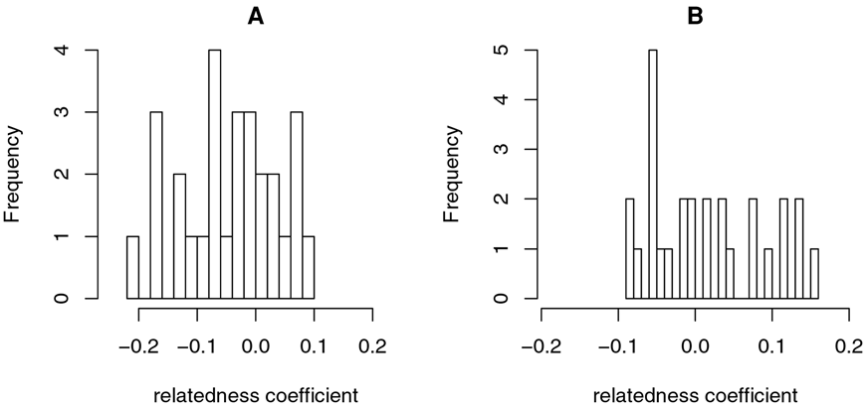
Distribution of relatedness coefficients across the MHC region among couples in the two samples. A) European American sample. B) African sample.

Using molecular markers (average relatedness coefficients across 9,010 SNPs), we observed that on average European American spouses were significantly more MHC-dissimilar from each other than random pairs of individuals (*R* = −0.043, *p* = 0.015). Moreover, the distribution of genetic relatedness coefficients across couples shows no outliers (Figure 1), thus excluding the possibility that this significantly negative coefficient could result from only a few couples having extremely low genetic relatedness. On the other hand, the MHC relatedness coefficient was positive but not significantly so in African couples (*R* = 0.015, *p* = 0.23). In addition, our analyses based on HLA types for 6 genes confirmed this broad pattern: the multilocus relatedness coefficient was marginally significantly negative in European American couples (*R* = −0.062, *p* = 0.084) and not significantly positive in Yoruba couples (*R* = 0.023, *p* = 0.412). (These analyses refer to the 4 digit classification. Similar patterns were seen with 2 digit classification; data not shown.) Using SNP data, we observed a higher mean SNP diversity in the MHC region in the African sample (0.366) than in the European American sample (0.349).

To control for genome-wide effects, we compared these observations to the pattern of genetic similarity across the genome (3,214,339 markers). Genome-wide, European American spouses were not significantly more or less similar than random pairs of individuals (genome-wide *R* = −0.00016, *p* = 0.739). On the other hand, African spouses were more similar genome-wide than random pairs of individuals (genome-wide *R* = 0.00185, *p*<10^−3^).

**Figure 2:**
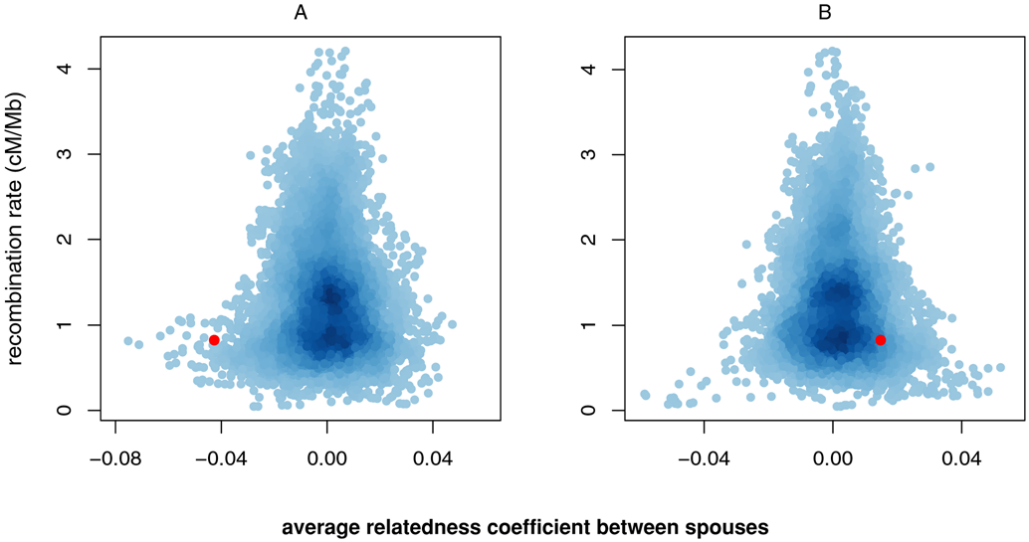
Average relatedness coefficients between spouses across overlapping 3.6 Mb regions throughout the genome, plotted against their recombination rate. The MHC is plotted in red. A) European American sample. B) African sample.

To further control for genome-wide effects, we asked whether the MHC region was unusual relative to similar regions across the genome with regard to its similarity/dissimilarity between spouses, by comparing the similarity between spouses at the MHC to that of all genomic windows having the same length as the MHC (3.6 Mb). Strikingly, in the European American couples, only 0.4% of the windows, concentrated in 9 genomic regions (listed in Table 1), exhibited a higher level of genetic dissimilarity between spouses than the MHC (Figure 2). To account for the particular linkage disequilibrium structure of the MHC and its low recombination rate [28], we compared the MHC region to a sub-set of windows having the same or lower recombination rate and still found that only 0.1% of these windows had less genetic similarity between spouses than did the MHC. In the African sample, 9% of the windows (and 17% when matching for the recombination rate) concentrated in 116 regions exhibited more genetic similarity between spouses than the MHC (Figure 2).

**Table 1:** Locations (in build 35 coordinates) of the nine regions exhibiting a higher level of genetic dissimilarity between spouses in the European American couples than the MHC.

Chr	start	end
1	168300000	171900000
3	78600000	83100000
4	27300000	30900000
4	149400000	154200000
6	116400000	120000000
10	100500000	106200000
12	46800000	51000000
15	40800000	47700000
17	53700000	57600000

## Discussion

At the molecular level, we found that the European American couples we studied are significantly more MHC-dissimilar than random pairs of individuals, and that this pattern of dissimilarity is extreme when compared to the rest of the genome, both globally and when broken into windows having the same length and recombination rate as the MHC. Our analyses based on HLA types also show a signature of dissimilarity between spouses. Such dissimilarity, observed from both molecular and serological data, cannot be explained by demographic processes, since such effects would affect the whole genome. On the other hand, this MHC dissimilarity could result from pressure for disassortative mating at the MHC level. Such a mechanism could be triggered by our olfactory capacity for discriminating MHC-mediated odour types [21],[29]. Alternatively, this genetic dissimilarity could result from selection of the spermatozoa by the female oocyte (post-copulatory sexual selection), a further safeguard favouring the production of MHC-heterozygous offspring more resistant to pathogens see [29]–[31] for reviews. Indeed, all studied couples were selected for having offspring, and the excess of dissimilarity observed could be restricted to fertile couples, rather than couples in general. However, further analysis showed that the offspring of these couples were not more MHC-diverse than expected by random selection of parental gametes (results not shown). Moreover, our results in European American couples reinforce previous evidence of MHC-disassortative mating among Hutterite couples [22], in which all couples were included, regardless of whether they had a child (C. Ober, personal communication). Like the Ober study, the sampled couples in our study are from a cultural isolate (in our case sampled from the Mormon community), so one might speculate that MHC-based mate choice is stronger or easier to detect in settings where there is less heterogeneity in other factors which influence mate choice, but the current absence of detailed molecular studies of mate choice in other human populations makes this impossible to assess. The two studies in Swiss males and females showing a significant preference of females for the odor of MHC-dissimilar (over MHC-similar) males [24],[25] implicate one possible mechanism by which couples may implement MHC-dependent mate choice. Taken together, these results strengthen the hypothesis that MHC genetic variation influences mate choice in some human populations.

Our analyses of the European American sample also show that the results based on molecular data were more significant than those based on HLA types. Although we cannot rule out power effects in explaining such a difference, it seems plausible, and consistent with our data, that the biological mechanisms involved in disassortative mating would depend on the MHC in ways that are not simply captured by HLA types. Such biological mechanisms could possibly result from a summation of effects over multiple genes, and not only from the six HLA genes studied here.

On the other hand, Yoruba couples exhibited a significant genome-wide signature of assortative mating, which is likely to result from socio-demographic processes specific to this population. The Yoruba are still organized in paternal lineages, which are exogamous units [32] and C. Adebamowo, personal communication. Although we do not have specific ethnological data collected with the Yoruba samples to explain our observations, a process in which matrimonial exchanges between genealogically related lineages are more frequent than matrimonial exchanges between genealogically unrelated lineages could have left such a genome-wide signature. On the contrary, for the MHC region, no significant pattern of similarity/dissimilarity was observed, at either the molecular level or the serological level. Several hypotheses can be proposed to explain this observation: firstly, it is possible either that the MHC is not involved in mate choice in this population, or that social factors are relatively more important than the MHC and that the sample size here does not allow detection of MHC effect on mate choice. Secondly, it is possible that MHC-based mate choice is aiming for an optimal, rather than maximal, number of MHC alleles previous theoretical and experimental evidence for this hypothesis are reviewed in [21]. Such a mechanism would explain why evidence of disassortative mating was found in the European Americans, all sampled in the Mormon community exhibiting a relatively low SNP diversity in the MHC (0.349), as well as in the genetically isolated Hutterite community [22], but not in Yoruba. Indeed, the Yoruba exhibit a relatively higher SNP diversity in the MHC (0.366) than the European American, and the optimization of the number of HLA alleles in Yoruba may involve mating with a not-so-MHC-dissimilar individual. This hypothesis is also consistent with the “sweaty T-shirts” experiment performed between females and males from different ethnicities (thus having a higher range of MHC dissimilarity than males and females coming from the same community) and showing that females prefer the odor of males with little MHC-dissimilarity than the odor of males with more extreme MHC-dissimilarity [26]. Finally, it is possible that in African populations, individuals carrying pathogen-resistant alleles are easier to identify than elsewhere, because of the higher pathogen pressure. In such conditions, it is possible that mating preferences for particular pathogen-resistant MHC alleles are stronger than mating preferences for MHC-dissimilarity per se [21].

In conclusion, our study, based on a large number of molecular markers which allow us to control for genome wide effects, indicates a clear-cut signature of MHC-disassortative mating in a sample of European American couples. This supports the existence of MHC-related biological factors contributing to mate choice in at least some human populations. On the other hand, the Yoruba exhibit a genome-wide tendency for enhanced similarity among couples but no significant pattern at the MHC level. This suggests that socio-demographic factors may be more important than biological factors for mate choice in this population, although the existence of MHC-dependent mate choice in Yoruba, aimed at optimizing (rather than maximizing) the number of HLA alleles in the offspring, cannot be excluded. Our study indicates that the relative importance of biological and social factors varies from one population to another. It also highlights the need for the exploration of further genome-wide data in larger sample sizes, including “just married” childless couples, sampled in several ethnically differentiated groups, in order to build a more robust view of the biological determinants acting on mate choice in humans.

## Materials and Methods

### Datasets

Two datasets were analysed in this study:

- 3,214,339 Single Nucleotide Polymorphism (SNPs) from the the Hapmap II dataset, typed in 30 European American couples (60 individuals) from Utah (Centre d'Etude du Polymorphisme Humain (CEPH) Collection) and 30 African couples (60 individuals) from the Yoruba population, Ibadan, Nigeria [27]. We used the phased data files and excluded SNPs with minor allele frequency below 5%. In this dataset, 9,010 SNPs were located in the MHC region, (positions 29,700,000–33,300,000 on chromosome 6 [19], in build 35 coordinates). Sex chromosomes were not included in the analyses.
- A set of HLA types for 6 of the main HLA genes (three class I genes: HLA-A, -B, -C, three class II genes: HLA-DQA, -DQB, DRB) in 44 European American couples and 30 African couples from the same collections as above [28]. 30 out of the 44 European American couples were shared with the HapMap II dataset, and all African couples were common to both datasets. This dataset is available on the following site: http://www.sanger.ac.uk/HGP/Chr6/.

### Relatedness Analyses

We estimated the genetic relatedness between spouses using SNP data and HLA type data. In all cases, the relatedness coefficient for a given pair of spouses *R* was defined as *R* = (*Q_c_*−*Q_m_*)/(1−*Q_m_*), where *Q_c_* is the proportion of identical variants between the two spouses and *Q_m_* is the mean proportion of identical variants in the sample (that is, averaged over all possible pairs of individuals) [33]. The proportion of identical variants at a given SNP for a given pair of individuals was 0 if both individuals were homozygous and carrying a different allele (eg 00 and 11), 1 if both individuals were homozygous and carrying the same allele (eg 00 and 00), and 0.5 in all others cases. We also considered a variation of this definition, with pairs of heterozygous individuals (01 and 01) being attributed a proportion of identical variants of 1 (instead of 0.5). Both definitions gave similar results (we present here coefficients based on the first definition). We estimated the average genetic relatedness coefficient between spouses across the MHC and across the whole genome. We checked that our estimates were not affected by the heterogeneity of SNP density and linkage patterns across the genome, by redoing our analyses on reduced sets of approximately independent SNPs prepared using two different procedures implemented in the software PLINK (one based on pairwise SNP r^2^ values and the other on the variance inflation factor) [34]. We also computed the average genetic relatedness coefficient between spouses for sliding windows of 3.6 Mb across the genome (in increments of 300 Kb) having at least 1,000 SNPs and not overlapping a centromere. In the case of the HLA type data, we defined the proportion of identical variants as 0 if the two individuals carried different types, 0.5 if one of their two types was similar, and 1 in all other cases and we computed a multi-locus relatedness coefficient between spouses based on types for 6 HLA genes. *R* was summarized across the MHC region, the genome or the six HLA genes by averaging *Q_c_* and *Q_m_* over all SNPs or over all HLA loci (and then computing the ratio (*Q_c_*−*Q_m_*)/(1−*Q_m_*)). Using molecular data, we also computed the mean SNP diversity (probability that two randomly chosen chromosomes are different at a given SNP) in the MHC region for both samples.

We removed from both datasets two European American and three African couples, in which one of the spouses had previously been found to be closely related (relatedness coefficient equal or higher to 1/32, see supplementary table 15 from [35]) to another sample (in each case, we chose at random the couple to be excluded). The relatedness coefficients before and after these exclusions were very similar (we present in the paper the estimates without these couples). In the case of HLA type data, we considered both the 4 digit and the 2 digit classification, and found congruent relatedness coefficients (coefficients based on the 4 digit classification only are reported in this paper). The significance of the relatedness coefficient was assessed using a permutation approach: the two-sided *p*-value is the proportion of permutations (attributing a new wife randomly to each husband) in which the permuted couples had the same or more extreme mean relatedness coefficient than the real couples. 1000 permutations were performed.
